# 多孔材料基吸附剂在环境和食品样品中提取农药残留物的最新应用

**DOI:** 10.3724/SP.J.1123.2024.12009

**Published:** 2025-07-08

**Authors:** Qitong XU, Meng YU, Chang XIE, Yan CAO, Surong MEI

**Affiliations:** 华中科技大学公共卫生学院，教育部环境与健康重点实验室，湖北 武汉 430030; Key Laboratory of Environment & Health of Ministry of Education，School of Public Health，Huazhong University of Science and Technology，Wuhan 430030，China

**Keywords:** 农药残留物, 多孔材料, 吸附剂, 前处理技术, 环境和食品样本, 综述, pesticide residues, porous materials, adsorbents, pretreatment technologies, environmental and foodstuff samples, review

## Abstract

近年来，全球范围内农药的使用量显著增长。随着各种新一代农药的陆续推出，其在环境中的分布特征也变得越来越复杂。因此，有必要开发快速、灵敏的多残留分析技术，以了解遗留和新型农药残留物在环境和食品介质中的分布状况。在分析过程中，样品预处理是一个不可或缺的环节，尤其是吸附剂的技术开发是关键因素。迄今为止，用于富集农药残留物的材料种类繁多。传统固相萃取（SPE）吸附剂已被广泛使用，但其缺乏特异性相互作用，选择性差。具有大比表面积和孔隙率的碳材料（如氧化石墨烯、碳纳米管）取得了一定进步，但其活性吸附位点仍然不足。而多孔材料，包括金属有机框架（MOFs）、多孔有机聚合物（POPs）、沸石（zeolites）以及多孔碳材料（NPCs）因孔隙率高、孔径可调、比表面积大和修饰位点丰富而显示出卓越的性能。在本综述中，我们首先介绍了提高多孔材料基吸附剂吸附性能的一些策略，包括材料杂化、单体改性、构型调制和表面性质调节；之后总结了2018年至今有关多孔材料基吸附剂用于各类农药富集的文献。文章主要讨论了农药的特性、多孔材料的设计思路和富集性能以及它们之间的相互作用机理。总体而言，研究人员主要根据目标农药的特性来设计吸附剂，以提高吸附性能和选择性。此外，我们还探讨了多孔材料基吸附剂的应用潜力，发现传统农药在萃取技术领域备受关注，而新型农药以及一些高频检出的农药所受关注还不够充分，在今后的吸附剂研究中应优先考虑这些目标物。

## Abstract (en)

Pesticides are ubiquitous to human life but their residues are indispensable micropollutants that threaten human health. In recent years， the global use of pesticides has increased significantly in recent years， and their environmental profiles have become increasingly complex as different generations of pesticides have appeared on the market. The residues of various legacy and emerging pesticides are omnipresent in both the environment and food medias. Consequently， developing rapid and sensitive detection technologies for analyzing multiple residues is imperative. Sample pretreatment， particularly adsorbent selection and innovation， is indispensable in this regard. So far， a wide range of hybrid nanomaterials have been used for the enrichment or adsorption of pesticide residues. While traditional solid-phase-extraction-based （SPE-based） sorbents are widely used， they lack specific interactions and are poorly selective. Normal carbon materials （e.g.， graphene oxide and carbon nanotubes）， which have large surface areas and pore volumes， have progressed significantly； however， they still have insufficient active adsorption sites. Notably， porous materials， including metal-organic frameworks （MOFs）， porous organic polymers （POPs） （including covalent organic frameworks （COFs）， covalent triazine frameworks （CTFs）， conjugated microporous polymers （CMPs）， microporous organic networks （MONs， sub-familied by CMPs， porous aromatic frameworks （PAFs）， and hyper-crosslinked polymers （HCPs））， nano-porous carbons（NPCs）， and zeolites display exceptional properties because they have high porosity， tunable pore sizes， large surface areas， and diverse modification sites. In this review， strategies for the enhancement of adsorption performance of porous-material-based adsorbents， including materials hybridization， monomer modification， configuration regulation， and properties adjustment are first introduced. Furthermore， publications from 2018 to 2024 pertaining to the utilization of porous-material-based adsorbents for diverse types of pesticides were briefly elaborated. The properties of pesticides， the designs and performance of porous materials， and their interaction mechanisms were discussed. A total of 14 types of pesticides are included in the discussion， namely organochlorine pesticides （OCPs）， organophosphorus pesticides （OPPs）， pyrethroids （PYRs）， benzoylurea insecticides （BUs）， neonicotinoid insecticides （NEOs）， phenyl-pyrazole insecticides （PPZs）， phenoxy carboxylic acid herbicides （PCAs）， triazine herbicides （TRZHs）， benzimidazole fungicides （BZDs）， azole/triazoles fungicides， strobilurin fungicides （SFs）， carbamate insecticides （Carbs）， phenyl-urea herbicides （PUHs）， and diamide insecticides. Our summary revealed that an adsorbent was predominantly designed based on the textural properties of the target pesticide and the structural characteristics of the hybrid material， such as its functional groups， polarity， and pore size， to enhance adsorption performance and selectivity. MOFs and POPs are the most commonly used pesticide adsorbents， whereas fewer NPCs have been reported in this regard. Additionally， the applications potentials of porous-material-based adsorbents were explored. The findings revealed that conventional pesticides， such as OPPs， have been significantly researched in the extraction technology field. In contrast， concerns surrounding newer pesticides， including NEOs， PPZs， and SFs， as well as some significantly detected residues （BZDs and TRZHs）， have not been fully addressed， highlighting the need for future adsorbent research that prioritizes emerging and significantly detected pesticides.

20世纪以来，合成农药（主要包括杀虫剂、除草剂和杀菌剂）已广泛应用于病媒控制^［1］^。由于农药不断地更新换代，传统农药和新型农药的总销量经历了几次逆转。20世纪40年代，有机氯类杀虫剂（OCPs）首先在美国使用，不久因其持久性和高毒性逐渐在许多国家被禁用^［2］^。之后，许多经典农药诸如有机磷类杀虫剂（OPPs）^［3］^、苯氧羧酸类除草剂（PCAs）、三嗪类除草剂（TRZHs）^［4］^、氨基甲酸酯类杀虫剂（Carbs）、苯并咪唑类杀菌剂（BZDs）和拟除虫菊酯类杀虫剂（PYRs）^［5］^取代了传统的OCPs。它们在全球农药市场中占据了很大的比例，其使用量从1990年到2019年甚至增长了1.5倍^［6］^。近年来，市场上出现了一些更新的农药，包括新烟碱类杀虫剂（NEOs）^［7］^、苯基吡唑类杀虫剂（PPZs）、双酰胺类杀虫剂（diamides）^［8］^，以及甲氧丙烯酸酯类杀菌剂（SFs）^［9］^。其使用量增长迅速，部分已超过一些经典的农药^［10，11］^。在中国的第五次到第六次总膳食研究中，NEOs和氟虫腈的检出水平和频率呈上升趋势^［12，13］^。与之相反的是，OPPs在日本的使用量逐渐下降^［14］^。从1998到2017年，OCPs在人体内的含量水平明显下降，从3 000~4 000 ng/g lipid^［15］^ 到500~600 ng/g lipid^［16］^。农药生产和使用历史的详细情况见图1。

**图1:**
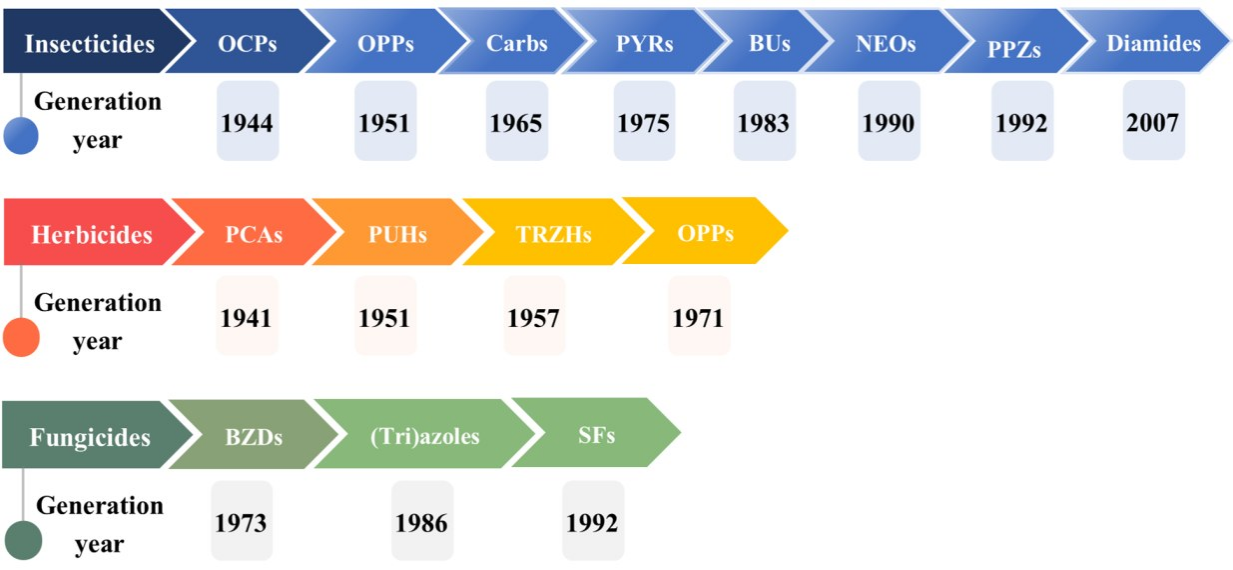
杀虫剂、除草剂和杀菌剂在全球市场上的发展和使用历史

农药的过度使用会导致其残留物迁移和累积到水体^［17］^、土壤^［18］^以及灰尘中^［9］^。残留在农作物和食品中的农药会引起严重的食品安全问题^［7］^。残留的农药最终会进入人体，造成广泛的人体暴露，导致各种不良健康结局^［19-21］^。尽管传统农药的环境残留问题长期存在^［22］^，但新兴农药已完成广泛登记注册，并在农业生产、兽医实践、工业运作及公共卫生保护等领域中并得到大规模应用^［23］^。因此，农药残留问题也变得日趋复杂。大量研究强调了不同类型的新兴农药和传统农药在环境中的不平衡残留现状^［11，22，24，25］^。另一方面，农药的不断更新换代是新型污染物逐步上升的重要原因。在此背景下，开发高灵敏，高选择性和高准确性的多残留方法，以监测环境和食品介质中农药的分布情况是十分必要的。

农药在样品中以痕量水平存在，样本中复杂的基质环境使得直接监测的困难较大。而样品前处理这一技术可解决此问题，通过从样品中提取和富集靶标，排除干扰成分，可显著提高分离效率^［26］^。在各种提取技术的研究中，探索适合不同目标分析物，且具有优异富集性能的吸附剂是一个关键的科学问题^［27］^。传统的固相萃取（SPE）吸附剂包括亲水亲油平衡吸附剂（HLB）、*N*-丙基乙二胺吸附剂（PSA），以及C_18_吸附剂，在多残留分析中得到了广泛应用^［28］^。尽管如此，这些商业吸附剂仍显示出一些局限性，例如缺乏特异性相互作用，导致富集不足和选择性差。当前，各类新型吸附材料在萃取和萃取领域备受欢迎。其中，多孔材料，包括金属有机框架（MOFs）、多孔有机聚合物（POPs）、多孔碳（NPCs）以及沸石，表现出独特的性能（大比表面积、高孔隙率、可调的孔径、丰富的修饰位点）以及优越的吸附性能和选择性^［29］^。它们具有可设计性，种类繁多，有望成为样品前处理和污染物去除领域中有潜力的吸附材料^［30］^。

近年来，相关综述报道了农药残留的预处理和移除的研究现状，其中一些文献仅针对一类多孔材料在农药残留中的应用进行总结^［31‒33］^，另一些文献着重讲述多孔材料在提取技术中的应用^［34］^。这些综述主要讨论了吸附材料的特性和制备方法，但很少对多孔材料基吸附剂的调控策略进行全面讨论。其他综述的关注重点集中在农药的类型、使用以及它们在环境中的分布情况^［1，6］^。然而，新型农药的关注度较少，所讨论的吸附剂主要是传统的碳基材料。相较之下，很少有文章对新型多孔材料基吸附剂在不同类农药残留的应用进行系统阐述。此外，考虑到不同农药在环境和人体中的残留/暴露水平差异，选择高频检出的农药进行吸附剂设计更具公共卫生意义。

因此，本文主要针对以下几部分进行综述：（1）介绍提升多孔材料吸附剂的吸附性能的设计策略；（2）对2018年至今发表的有关多孔材料基吸附剂在环境和食品样品应用于各类农药的文献进行总结；（3）讨论多孔材料基吸附剂在不同类型农药中的应用潜力。

## 1 多孔材料基吸附剂的概述及提高其吸附性能的策略

新型多孔材料表现出有序的网络骨架，可调的构象，多样的官能团和大量的吸附位点^［29］^。他们可以被分为有机多孔材料（如POPs）、无机-有机杂化多孔材料（如MOFs）以及无机多孔材料（如沸石和NPCs）。具体而言，POPs包括共价有机框架（COFs）、共价三嗪框架（CTFs）、共轭微孔有机物（CMPs）、微孔有机网络（MONs）、多孔芳香骨架（PAFs）和超交联聚合物（HCPs）（图2）^［35］^。其中，COFs是通过缩合反应由有机单体构成的经典POPs。HCPs、MONs和CMP等则是由特定反应如Friedel-Crafts烷基化反应和Sonogashira偶联反应^［43］^合成的相对较新的POPs。不同类型的多孔材料具有不同的特性。MOFs是由金属团簇和多功能有机连接物构成的配位聚合物，特殊的金属团簇赋予其额外的不饱和开放金属位点和可调的孔径，使得介孔和微孔共存于其中^［44］^，而POPs则是通过共价键构建而成，由于孔径的限制，大部分吸附行为只发生在其表面，内部孔隙利用率较低^［45］^。然而，由于其内部牢固的共价键，POPs具有很强的热稳定性和化学稳定性。相比之下，具有配位键的MOFs稳定性较差，易受酸、碱和热的影响。可以根据多孔材料和目标分析物的特点对多孔材料基吸附剂进行设计和修饰。

**图2:**
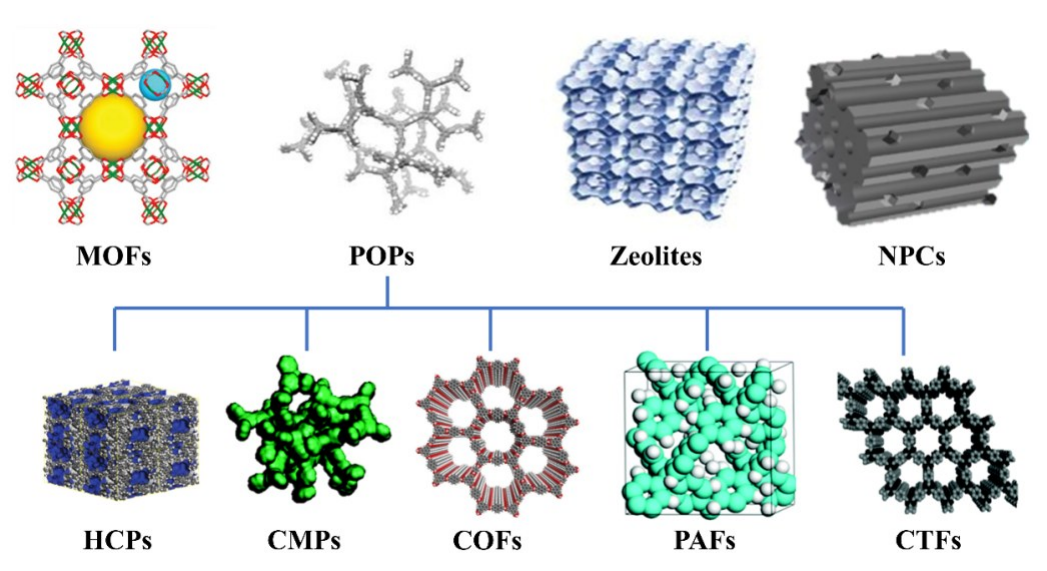
不同类型多孔材料的结构模拟图，包括MOFs^［36］^、POPs^［37］^、Zeolites^［37］^、NPCs^［38］^、HCPs^［39］^、CMPs^［40］^、COFs^［32］^、PAFs^［41］^和CTFs^［42］^

没有一种单一的多孔材料可以同时满足多种功能，研究人员设计了混合或多功能吸附剂来发挥互补作用。截至目前，各种杂化多孔材料，如氢键有机框架^［46］^、MTV-MOFs（多元MOF，具有多种类型的功能单体）^［47］^、聚二乙烯苯^［48］^以及MOF/COF 衍生材料^［49］^ 已被用于污染物的吸附。几种材料设计的主要策略如图3所示。

**图3:**
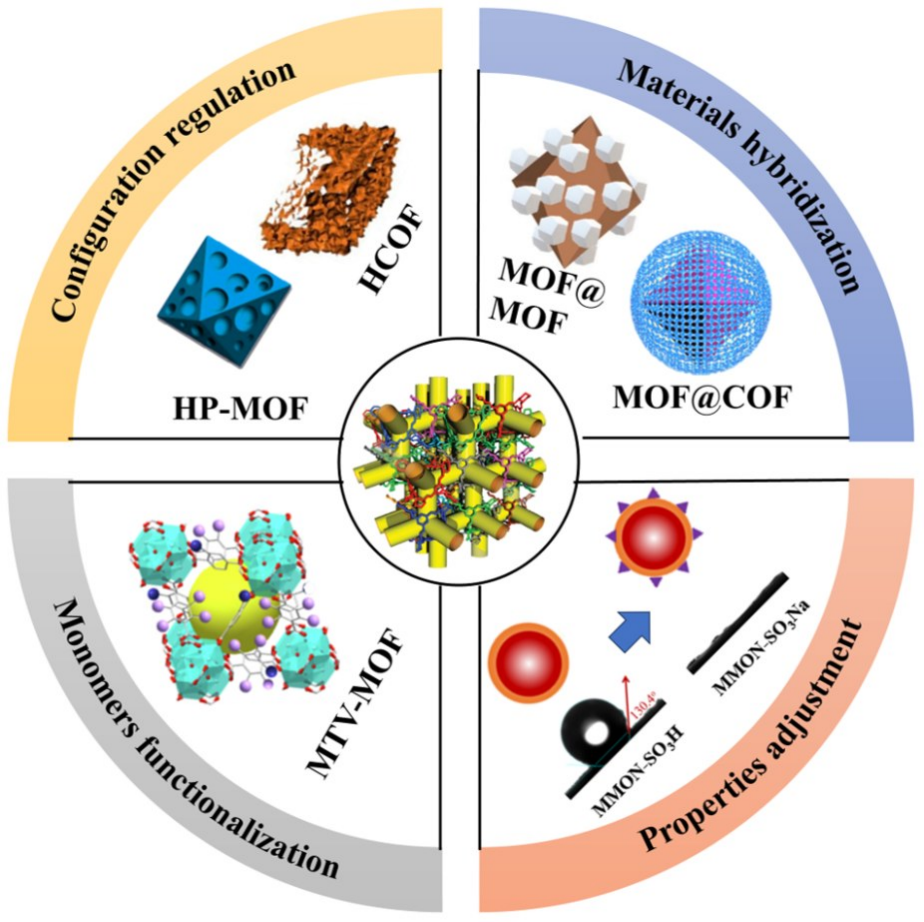
多孔材料基吸附剂提高萃取性能的主要改性策略

### 1.1 材料杂化

构建复合材料基吸附剂是移除/萃取技术领域的常用手段。几种杂化或多功能多孔材料基吸附剂已经被开发出来，包括双MOF^［50，51］^，MOF@COF^［52］^以及其他多孔材料基复合材料^［53］^。Zhou等^［54］^将磁性碳纳米管（MCNT）与ZIF-67相结合，用于吸附水体中的四溴双酚A，MCNT优异的机械强度可改善MOF不稳定的特点，其大比表面积也有利于ZIF-67的生长。

Yuan等^［55］^制备了 MIL-53（Fe）/ZIF-8用于去除抗生素。其中，ZIF-8对靶向抗生素具有良好的亲和力，但容易发生团聚，大尺寸的MIL-53（Fe）起到了支撑作用，使ZIF-8在其表面均匀生长，ZIF-8则弥补了MIL-53（Fe）再生能力差的缺陷。在批量吸附实验中，MIL-53（Fe）/ZIF-8的吸附性能显著优于MIL-53（Fe）和ZIF-8。Selahle等^［56］^将ZIF-67和磁性多孔有机聚合物（M-PPOP）整合，作为吸附剂用于富集NEOs。二者的结合使得该复合材料具有更高的比表面积和对NEOs优异的吸附亲和力。

### 1.2 单体功能化

在孔环境中引入官能团是提高多孔材料对目标化合物亲和力和选择性的有效策略，通常通过合成后修饰和调制法来实现。例如，Yang等^［57］^合成了一种名为2D-COF-CN（2D： 二维）的功能化COF作为OCPs的固相微萃取剂。氰基的接枝为吸附剂提供了更多的活性位点。Negro等^［58］^制备了一系列硫醚基MTV-MOFs，用于吸附NEOs。通过系统控制不同功能单体的比例，他们确定了具有最优捕获性能的MOF。Zhang等^［59］^构建了一系列接枝不同长度的胺功能化碳链的COFs，用于吸附双氯芬酸钠。此方法能同时做到孔径的调节和亲和基团的引入。研究表明，COFs的吸附能力不仅受孔径大小的影响，还受氨基存在的影响。Zhou等^［60］^以4-甲酰苯甲酸为调制剂，合成羧基功能化COF，用于萃取极性硝基苯化合物。相较于后合成修饰法，调制法引入官能团更高效，难易程度更小。

### 1.3 构型调控

孔隙填充效应是吸附过程中的重要驱动力，需要吸附剂的孔道与目标分析物尺寸匹配。许多研究人员通过构建分层孔隙或缺陷位点，开发出具有合适孔径的多孔材料基吸附剂。Li等^［61］^以Cu-MOF作为牺牲模板，开发了一种分级孔COF，用于食品安全非靶向分析的样品前处理。与此类似，他们又以ZIF-8作为牺牲模板，制备出一种中空COF，用于富集食品中的有害物质^［62］^。COF中共存的宏/中/微孔可以降低目标物的传质阻力，提高传质效率，增强内部孔隙利用率。Cao等^［63］^利用缺陷形成策略系统地定制了一系列不同尺寸孔径的Zr-MOFs。他们研究了不同孔径分布的MOF吸附性能与不同大小的目标分子之间的关系，发现MOF中的介孔结构可以解决竞争性相互作用的问题，使其适用于多组分吸附。

材料形貌的改变也会显著影响其吸附性能。粒径较小、比表面积较大的多孔材料往往能获得较好的吸附效率^［64］^。Xiao等^［65］^开发了一种三维Al-TCPP（TCPP：四（4-羧基苯基）卟啉）纳米片，用于捕获水中的氟虫腈，并将其吸附性能与相应的块状晶体进行了比较。结果表明，分层多孔结构的Al-TCPP纳米片具有更优异的去除效率。

### 1.4 表面性质调节

调整吸附剂的表面性质是增强其与目标化合物相互作用的有效途径。依据“相似相溶性”，对吸附剂的亲疏水性进行调控，可实现对相应极性/非极性化合物的高效吸附。Wang等^［66］^制备了一系列极性可调的HCPs用于富集黄曲霉毒素。他们选择以烷基酚聚氧乙烯酸酯（OPs）作为单体，其含有不同长度的聚氧乙烯酸酯（EO）链，EO链的长度影响OPs的亲水性，从而间接影响HCPs的亲水性。结果表明，以含有10个EO链的OPs合成的HCPs具有合适的极性，对黄曲霉毒素有较好的提取效果。Zhou等^［67］^制备了一种名为TAPB-DMTP-DB^a^ （TAPB：1，3，5-三（4-氨基苯基）苯；DMTP：2，5-二甲氧基对苯二醛；DB^a^：2，5-二甲氧基苯甲醛）的COF。通过引入不同比例的DB^a^可调节其疏水性。随着DB^a^比例的增加，COFs含有更丰富的甲氧基，拥有更高的疏水性和比表面积。实验结果表明，含有40%DB^a^的COF对多溴联苯具有最优的富集性能。

对于离子型分析物，可设计表面带电的吸附剂，通过静电相互作用增强亲和力。PCAs和BZDs是两种典型的离子型农药。Liu等^［68］^开发了一种名为TpTG_Cl_（Tp：1，3，5-三甲酰间苯三酚，TG_Cl_：三氨基胍氯）的阳离子COF，它为阴离子PCAs的快速捕获提供了大量的阳离子位点。Zhao等^［69］^制备了一种含有离子基团的两性POP，名为Ni/CTF-SO_3_H，用于食品样本中多菌灵（CBZ）和噻菌灵（TBZ）的选择性提取。在酸性环境下，Ni/CTF-SO_3_H上的磺酸基团可以通过阳离子交换作用与阳离子CBZ和TBZ相互作用。

## 2 用于农药吸附的多孔材料基吸附剂

### 2.1 多孔材料基吸附剂的应用潜力

农药作为一类新兴污染物，其控制和管理日益受到重视^［70］^。迄今为止，随着农药不断地更新换代，市场上陆续出现不同世代的农药^［70］^。其中，有相当数量的新型农药对生态环境和人体健康构成风险，但未被充分纳入管控范围^［71］^。因此，加强对这些新兴农药的环境监测和吸附剂开发至关重要。

为了调查吸附剂开发在农药方面的应用潜力，我们根据附表S1（见www.chrom-China.com）对14种农药的多孔材料基吸附剂的发表量进行了归纳总结（图4）。从2018年至2024年，通过在Web of Science和ScienceDirect数据库输入关键词“adsorbents”和“pesticides”进行文献检索，共纳入了196篇文章，其中传统或典型农药最受关注，而针对一些新型和高频检出农药的吸附剂开发相对有限。具体而言，OPPs是最受关注的农药，累积42篇文献报道了针对它们的吸附剂设计情况。其次是NEOs，由于其近年来的广泛使用而成为农药分析的热门目标物。尽管OPPs（特别是毒死蜱）在人体和环境样本中被广泛检测并显示出相对较高的水平，但它们正在逐渐被新一代农药所取代^［14］^。相反，NEOs在环境和人体中的检出水平呈上升趋势^［13］^，但其报道的文献数量仅为OPPs的一半。其他新型农药包括PPZs（典型PPZs： 氟虫腈）、SFs（典型SFs： 嘧菌酯）以及联胺类杀虫剂也得到了大力推广并在环境中被高频检出^［9，72］^。然而，针对这些农药的吸附萃取技术的开发很少。一些其他的高频检出农药（例如BZDs^［73］^和TRZHs^［74］^）在吸附领域的研究也较为少见。这些结果表明，当前针对新型和高频检出农药的关注较为有限，研究人员需要将注意力从传统农药转移到这些更有开发前景的农药上，加强其在未来吸附领域的研究。

**图4:**
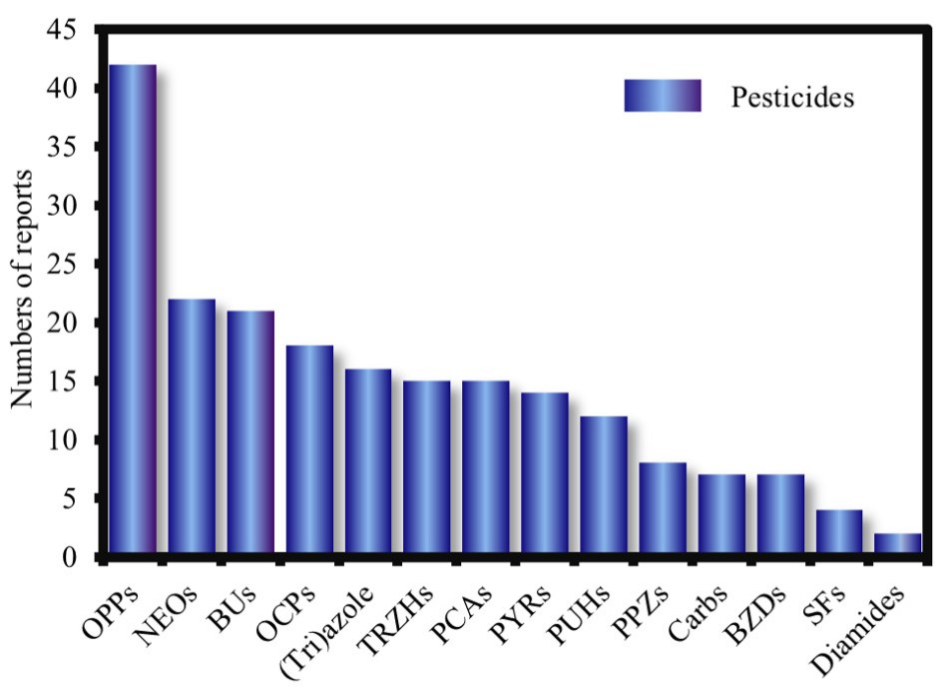
2018-2024应用于不同类型农药的多孔材料基吸附剂文献数量

### 2.2 用于农药吸附的多孔材料基吸附剂开发进展

当前，多种多孔材料基吸附剂已被设计开发，用于不同家族农药的富集。大多数农药是含氧或含氮基团的芳香族化合物，部分含有卤素基团^［75］^。多孔材料具有*π*-电子共轭体系和多种官能团，可与目标农药形成*π*-*π*堆叠、氢键和偶极-偶极相互作用等。由于同一类农药具有结构类似性（共同的官能团、相近的极性和分子尺寸）^［1］^，因此许多研究致力于开发能同时提取同一类农药的吸附剂^［76-78］^。

对环境和食品样品中14种农药的多孔材料基吸附剂开发情况总结见附表S1。下面将介绍各类农药的性质、吸附剂的设计思路和特点、吸附剂的性能及其相互作用机理。

#### 2.2.1 OCPs

OCPs是非极性化合物，具有较高的log *K*_ow_值（3.70~6.44）（*K*_ow_： 正辛醇/水分配系数）^［79］^。因此，设计疏水性吸附剂是对其进行萃取的有效途径。Ma等^［79］^制备了PAN@COFs（PAN： 电纺纳米纤维）并研究了其与OCPs的疏水和氢键相互作用（图5）。PAN@COFs的提取性能与OCPs的log *K*_ow_值呈正相关，PAN的加入是为了提高COF在水相中的分散性。与此类似，Sun等^［53］^将*β*-环糊精与MOF整合以增强其在水中的相容性。*β*-环糊精具有亲水表面和疏水腔，使得该杂化材料在水中分散性良好且对 OCPs具有高亲和力。Guo等^［80］^合成了OAMPCs（含氮多孔碳材料），其水接触角（疏水性表征参数）在吸附完OCPs后增加，吸附容量达到了751.6 mg/g。OCPs含有大量氯化烃和共轭基团（氯化芳环和环己烷）。据此，Li等^［81］^和Yang等^［82］^分别在COFs中引入氰基和硼酸基团，用于OCPs的提取，这两类基团都可以为OCPs的有效捕获提供丰富的氢键和卤素键位点。

**图5:**
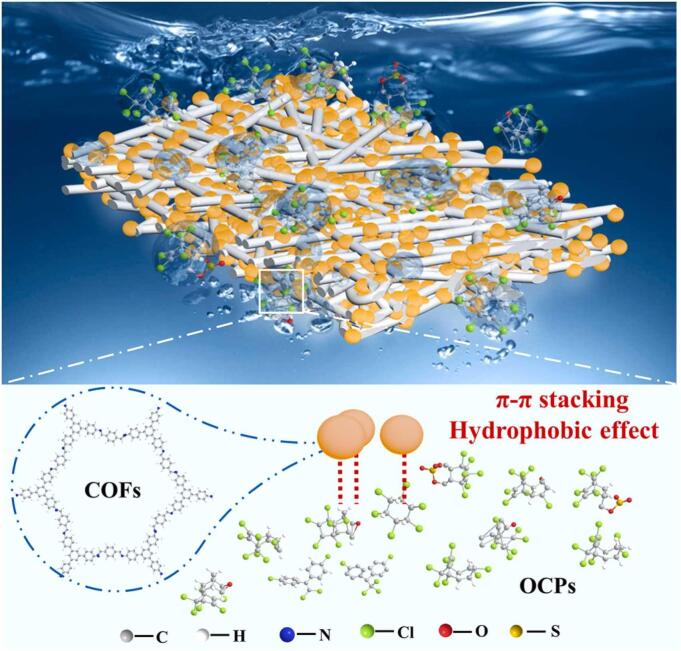
COFs 和 OCPs之间可能的吸附机理^［79］^

#### 2.2.2 OPPs

大多数OPPs是磷酸酯衍生物^［83］^。磷酸基团在OPPs中作为路易斯碱，它们可以与Zr-MOFs中的Zr_6_O_4_（OH）_4_和Zr-OH等独特的路易斯酸位点形成较强的亲和力。据此，一系列锆基多孔材料被开发以用于OPPs的吸附。Pankajakshan等^［84］^合成了两种Zr-MOF，分别是 NU-1000和UiO-67，并研究了它们对水中草甘膦的捕获效率。研究发现，NU-1000的效率高于UiO-67，这主要是由于它们的结构差异。NU-1000孔道较大，孔径为3.1 nm，而UiO-67孔道大小为1.4~1.6 nm。NU-1000中较宽的孔径有利于草甘膦与金属节点的相互作用。Li等^［85］^将Zr^4+^固定在COF表面，制备了Fe_3_O_4_@COF@Zr^4+^用于同时富集8种OPPs（附图S1，www.chrom-China.com）。为了改进UiO-67，Fang等^［86］^制备了一种缺陷型UiO-67用于吸附草甘膦，其最大吸附容量达到322.58 mg/g（图6）。缺陷的存在使得MOF内部形成许多分层多孔结构，并增加了自由基团（如Zr-OH）的数量。Fang等^［87］^将UiO-66-（OH）_2_与二乙烯基苯-*n*-乙烯基吡咯烷酮微球结合，合成了名为DVB-NVP@UiO-66-（OH）_2_的复合材料，为茶泡液中OPPs的提取提供了亲水-亲油平衡。

**图6:**
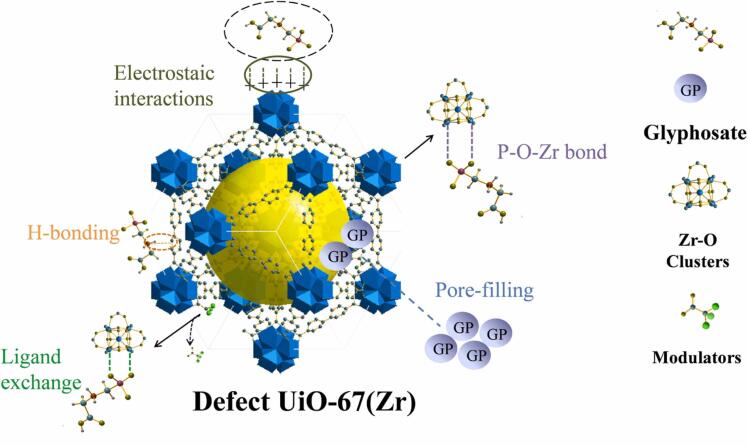
缺陷型UiO-67（Zr）和草甘膦之间可能的吸附机理^［86］^

除了金属Zr外，金属Ti和Zn也具有与磷酸基团络合的能力。Yang等^［88］^制备了NH_2_-MIL-125（Ti）基滤纸膜用于提取菠菜中的3种OPPs，最大吸附容量达到640 mg/g（附图S2）。一系列的Zn-MOF^［89-91］^用于OPPs的提取，并表现出较高的萃取效率。OPPs中的O和N原子可以作为氢键供体，与氨基或羟基形成氢键。基于此，Liu等^［92］^应用NH_2_-MIL-101（Fe）有效吸附草甘膦，最大吸附容量达到431.13 mg/g。Du等^［93］^设计了一种名为JNU-6的3D COF-OH（3D：三维），用于提取4种OPPs，其富集能力显著高于二维无羟基官能团的COF-300。

#### 2.2.3 PYRs

PYRs是一组疏水芳香族化合物（log *K*_ow_ 值为4.53~7.00），通过酸和醇发生酯化反应而合成^［94］^。疏水效应可能在吸附过程做出重要贡献。Wu等^［95］^制备了Fe_3_O_4_@NU-1000用于提取PYRs。吸附机理研究表明，疏水相互作用将化合物驱动到NU-1000表面，*π-π*和*p-π*相互作用有助于稳定NU-1000微孔中的PYRs。Yu等^［96］^报道了一种亚胺键连接COF涂层，该涂层具有较高的疏水性，适用于PYRs的提取，并得到较高的富集因子（EFs）（2 700~13 195）。在另一项研究中^［97］^，疏水离子液体修饰的磁性ZIF-8对PYRs表现出优异的富集性能。该材料具有较宽的孔径分布范围（1.7~300 nm），中孔和大孔共存，能够容纳相对分子质量较大的PYRs（相对分子质量：400~700）。He等^［98］^合成了Fe_3_O_4_-NH_2_@MIL-101（Cr）用于富集5种PYRs。MIL-101（Cr）的疏水通道足够大，可以让PYRs分子进入并通过*π-π*相互作用被吸附。PYRs携带的氰基、卤素和其他富电子基团很容易接近Cr（Ⅲ）的不饱和配位点。PYRs与磁微球中的氨基可以形成氢键相互作用。与此类似，由Yan等^［99］^开发的CTFs/Co复合材料中的氮原子以及由Jia等^［100］^设计的ATP@COFs（ATP： 凹凸棒石）中的氨基也能与PYRs形成氢键。一些已报道的MOFs模板多孔碳^［101，102］^均表现出对PYRs的高富集效率，这是由于它们丰富的疏水位点和促进*π*-*π*相互作用的*sp* ^2^-杂化碳结构。

#### 2.2.4 苯甲酰脲类杀虫剂（BUs）

作为第三代杀虫剂，BUs是由两个芳香环通过尿素桥连接而成的含氟化合物，为疏水化合物，log *K*_ow_值的范围为3.10~6.27^［103］^。除*π-π*堆积外，疏水和氟-氟相互作用是常见的吸附机制。一些氟化POPs^［104-107］^以及三氟甲基接枝的COF^［108］^（附图S3）被设计出来用于捕获BUs。Jia等^［109］^制备了Fe_3_O_4_@MOF-808作为吸附剂用于7种BUs的磁固相萃取（MSPE）。EFs值的排序与BUs的log *K*_ow_值排序相一致，表面疏水效应在萃取过程起重要作用。Xin等^［110］^制备了NH_2_-Fe_3_O_4_@COF用于茶饮料中6种BUs的萃取。COFs中的氧原子与BUs中的氟原子可以形成强的卤素键。由于BUs富含氮原子，Cui等^［111］^合成了羧基苯硼酸基官能化MOF，命名为CPBA@UiO-66@Fe_3_O_4_，用于5种BUs的提取，该材料可以通过B-N配位和*π*-*π*堆积与BUs相互作用。

#### 2.2.5 NEOs

NEOs是尼古丁的结构衍生物^［112］^。它们是一类小分子高水溶性化合物（相对分子质量约为200~300，log *K*_ow_值的范围为‒1.16~0.62）^［113］^，且含有丰富的电负性基团（氰基、硝基和卤素基）^［26］^。Xu等^［114］^应用NH_2_-UiO-66萃取食品样本中的吡虫啉（IMI）和噻虫嗪（THM），吸附容量达到373 mg/g，NH_2_-UiO-66中的氨基可以与IMI和THM形成氢键。与此类似，Ma等^［30］^设计了一系列水稳定的MIL-101衍生纳米材料用于富集水中9种NEOs（附图 S4）。其中，具有中等亲水性的NH_2_-MIL-101对9种NEOs具有普遍较高的亲和性。考虑到NEOs的强极性，几种亲水性多孔材料也被设计出来。Lu等^［115］^制备了Fe_3_O_4_@COF-（NO_2_）_2_用于提取蔬菜中的NEOs。与未改性的Fe_3_O_4_@COF相比，Fe_3_O_4_@COF-（NO_2_）_2_具有更高的亲水性和更好的吸附性能。其他以羟基功能化单体制备的亲水性MOF^［116］^和POPs^［113，117，118］^也被应用于NEOs的富集，羟基官能团赋予材料高亲水表面和大量氢键位点，促进了吸附过程的进行。此外，NEOs富含丰富的芳香杂环（吡啶和噻唑）作为*π*电子给/受体可以与多孔材料形成*π*-*π*电子供受体（EDA）相互作用^［38］^。

#### 2.2.6 PPZs

近年来，PPZs特别是氟虫腈及其代谢物在农业市场上迅速发展。当前，已报道部分含氟的多孔材料用于PPZs的萃取。Zhang等^［119］^合成了一种具有氟亲和性的磁性COFs，名为Fe_3_O_4_@COF-F。在它吸附PPZs的过程中，氟-氟和疏水相互作用占主导作用。Yang和Xia等^［120］^设计了一种三氟甲基修饰的分级孔MOF，名为BHP-Fe-CF_3_，用于去除3种含氟杀虫剂，包括氟虫腈（图7）。吸附过程涉及亲氟相互作用，孔隙填充和*π-π*堆积作用，最大吸附容量可达564.9 mg/g。Ba等^［121］^建立了一种基于CTF的前处理方法用于富集鸡蛋中的氟虫腈和它们的代谢物。氟虫腈及其代谢物具有丰富的苯/杂环和吸电子基团（氟、氯、氰），CTF中的杂原子环和噻嗪环可以与之形成*π-π*和氢键相互作用。

**图7:**
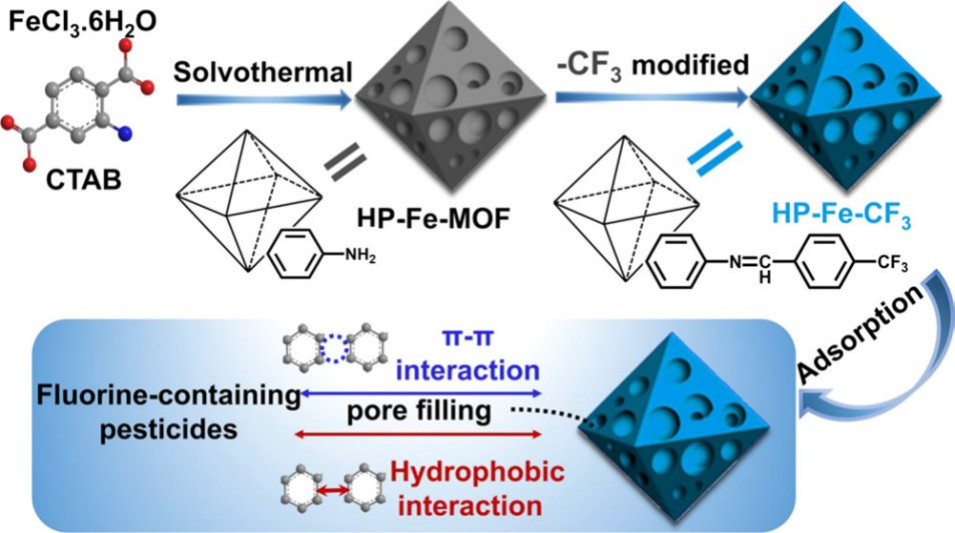
HP-Fe-CF_3_ 的合成及其和氟虫腈之间可能的相互作用^［120］^

#### 2.2.7 PCAs

PCAs作为一类小分子化合物（相对分子质量约为200~300），是一种离子型除草剂。它们主要以盐的形式存在于水溶液中^［122］^。在对PCAs的吸附过程中，静电相互作用是主要驱动力。Wu等^［123］^对UiO-66-NH_2_进行了电离改性，设计出了一种离子交换型MOF，名为UiO-66-NMe^3+^，用于2，4-D的去除，获得了279 mg/g的最大吸附容量，远高于Li等^［124］^制备的UiO-66-NH_2_/sponge（73 mg/g）。Liu等^［68］^开发了一种名为TpTG_Cl_的阳离子 COF，该COF拥有大量的阳离子位点，可通过静电相互作用实现对阴离子PCAs的快速捕获（附图S5）。尺寸匹配是有效吸附的重要条件。UiO-66-NH_2_的孔径（0.8 nm）略小于PCAs的分子尺寸（0.80~0.89 nm），导致吸附只发生在表面。而Liu等^［125］^则引入了Fe-Zr掺杂的MIL-101，其孔径更大，吸附容量达到357.14 mg/g。PCAs的苯环上具有羧基和丰富的亲电氯基团，一些文献据此设计出吸附剂通过氢键和卤素键与之相互作用^［126，127］^。

#### 2.2.8 TRZHs

TRZHs是一种含氮的芳香分子，具有C_3_H_3_N_3_杂环。它们属于中等疏水性小分子化合物（相对分子质量在200左右，log *K*_ow_为2~3）^［128］^。Guo等^［129］^制备了一种基于*β*-环糊精的磁性HCPs，用于萃取4种TRZHs。*β*-环糊精具有疏水空腔和丰富的羟基，可提供疏水和氢键相互作用。Liu等^［130］^设计了一种OH-MON作为SPE吸附剂用于6种TRZHs的高效富集（图 8）。密度泛函理论（DFT）表面氢键相互作用在富集过程起关键作用，而*π-π*、疏水相互作用和卤素键起辅助作用。具体而言，含烷基硫的TRZHs主要通过-OH⋯N-和-NH⋯O-相互作用，而含氯的TRZHs主要通过-O⋯Cl-相互作用。Li等^［131］^制备了一种基于咔唑单体的POP，命名为Car-DMB，用于富集食品样品中的4种TRZHs。根据DFT计算结果，发现强氢键和弱*π-π*堆叠是主要的相互作用。

**图8:**
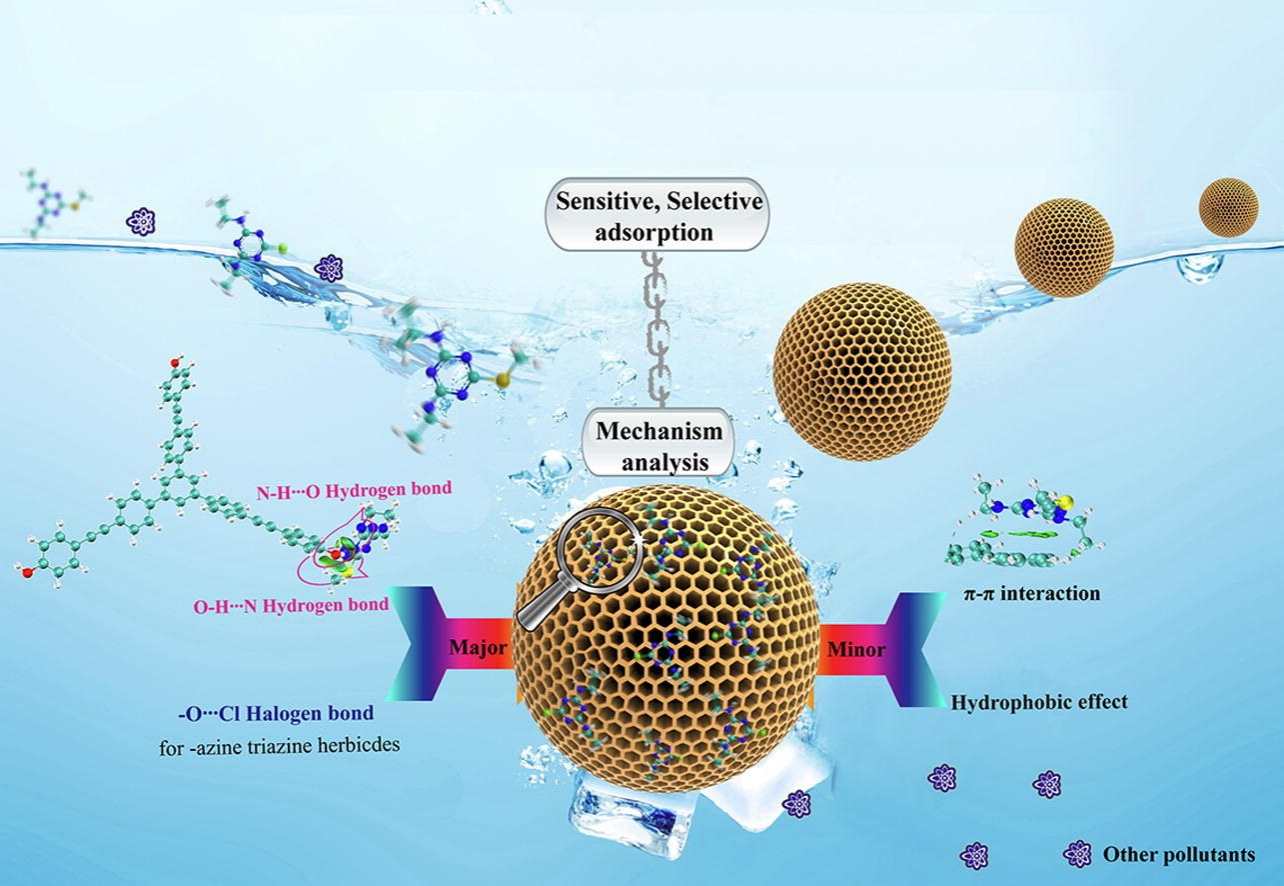
OH-MON 和 三嗪类除草剂之间可能的吸附机理^［130］^

#### 2.2.9 BZDs

作为一类杀菌剂，BZDs是由苯、咪唑环和酰基氨基构成的芳香杂环化合物^［132］^。这类化合物碱性强，在酸性环境下容易质子化（p*K*_a_为5~6）。Zhao等^［69］^制备了一种Ni掺杂CTF-SO_3_H，用于萃取食品样本中的CBZ和TBZ。He等^［133］^在此基础上做出改进，引入了一种富含磺酸钠基团的磁性MON，命名为MMON-SO_3_H@SO_3_Na，用于蔬菜和水果样品中4种BZDs的富集（图S6）。结果表明，与MMON-SO_3_H相比，MMON-SO_3_H@SO_3_Na表现出更好的萃取效率，这是由于亲水的磺酸钠基团可以促进阳离子交换过程。Xu等^［134］^运用UiO-67@GO@Fe_3_O_4_（GO： 氧化石墨烯）建立了一种MSPE方法用于富集蜂蜜中的BZDs。Zr-O键能与BZDs的硝基和氨基形成氢键。Sun等^［135］^合成了一种硅/氟-PAFs用于提取菠菜中的CBZ和TBZ。在PAF骨架上引入氨基基团显著增强了其对目标物的亲和力，吸附容量在30~40 mg/g左右。

#### 2.2.10 唑类/三唑类杀菌剂（Azole/Triazoles）

Azole/Triazoles是含有至少1个或3个含氮吡咯环的化合物，具有中等疏水性 ^［136］^。除了氢键和*π-π*堆叠作用外，研究人员还讨论了MOF中活性金属簇位点与唑/三唑分析物之间形成的共价键。Lu等^［137］^制备了Fe_3_O_4_@LS@ZIF-8（LS：木质素磺酸盐）用于去除水中两种唑类杀菌剂（图9）。该研究的表征结果证实了在吸附过程中ZIF-8中的Zn-O活性位点与杀菌剂分子之间形成了稳定的共价键合结构。Zhou等^［138］^合成了Fe_3_O_4_@ZIF-90@MIL-68（Al）复合物用于去除5种唑类杀菌剂。与此类似，此研究也探究了Al-O与分析物之间的共价相互作用。此外，MIL-68（Al）的网络结构中存在两种不同大小的孔道，既可以提高内部孔隙的利用率，又可以提高传质速率，针对咪鲜胺的最大吸附容量达到352.32 mg/g。Lu等^［139］^将双金属有机框架（Fe/Mg-MIL-88B）与磁性麦秆生物炭（MBC）相复合，用于吸附水中的唑类杀菌剂，Fe/Mg-O与目标物之间也形成了共价键。

**图9:**
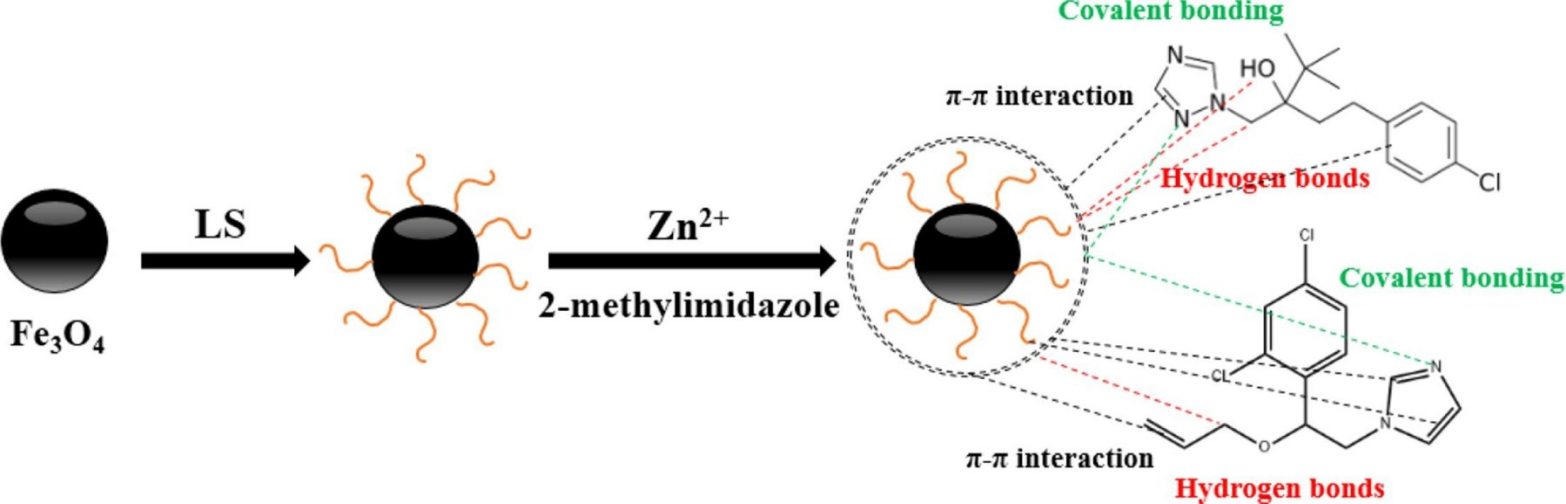
Fe_3_O_4_@LS@ZIF-8 和唑类杀菌剂之间可能的吸附机理^［137］^

#### 2.2.11 SFs

SFs是一种以strobilurin A（*β*-甲氧基丙烯酸酯）为先导化合物的仿生杀菌剂^［140］^。它们表现出疏水性，log *K*_ow_值为3.10~5.54。Wang等^［76］^应用一种名为Fe_3_O_4_@BTA-TAPM（BTA：联苯-3，3′，5，5′-四碳醛；TAPM：三（4-氨基苯基）甲烷）的磁性COF用于富集食品中的11种SFs。该材料具有大的离域*π*键和丰富的氮元素，这使其对目标SFs具有高亲和力。其最大吸附量为42.84~54.18 mg/g。Li等^［141］^合成了TAPT-Tp@Fe_3_O_4_（TAPT：1，3，5-三（4-氨基苯基）三嗪）作为MSPE吸附剂用于中草药中5种SFs的监测，表现出良好的分离性能。该COF骨架由芳香环和三嗪环构成，具有丰富的富电子官能团作为氢键位点参与吸附过程。

#### 2.2.12 其他农药

Carbs是一种氨基甲酸衍生的杀虫剂^［142］^。此类化合物通常具有富电子芳香环结构，如氨基、亚胺和羰基，可作为氢键作用的供受体位点。Gamonchuang等^［143］^基于苯甲酸酯配体设计了一系列MOF复合材料，并成功应用于7种Carbs的富集。苯甲酸酯配体中的羧基是一个强吸电子基团，可以降低芳环的电子密度，有利于配体与Carbs之间的EDA相互作用。Guo等^［144］^合成了COF-TpDB^b^（DB^b^：4，4′-二氨基苯甲酰苯胺）用于水果中4种Carbs的提取，相互作用机理主要为氢键^［145］^。苯基脲除草剂（PUHs）是一种水溶性较低的除草剂，含有丰富的氢键位点。Han等^［144］^合成了一种羧基功能化的MON（MON-2COOH），并将其作为搅拌棒涂层用于富集4种PUHs。研究比较了MON、MON-COOH以及MON-2COOH的萃取效率，其值随着羧基数量的增加而增加，这表明MON-2COOH与PUHs之间O⋯H-N相互作用的重要性。双酰胺类杀虫剂是一类含有酰胺键的新兴农药。Ma等^［146］^评价了7种代表性MOFs对双酰胺类杀虫剂的亲和力，发现MIL-101-NH_2_在这些材料中表现最好。另外，一种分级孔Al-TCPP纳米片被设计用于氯虫苯甲酰胺的萃取^［65］^。

## 3 总结与未来展望

在这篇综述中，我们概述了多孔材料基吸附剂及其优化策略，并着重介绍了其在农药预富集/去除中的应用。本文总结的内容对今后吸附剂的设计和开发提供重要参考价值。基于现有研究成果，提高吸附剂对农药残留提取能力的策略主要涉及材料杂化、单体功能化、构型调控和表面性质调节。得益于这些功能化策略，各种杂化多孔材料已被应用于不同种类农药的吸附。尽管取得了这些成就，在开发多孔材料基吸附剂的过程中仍面临着一些科学挑战。首先，传统的MOFs，例如UiO、ZIF以及MIL系列得到了大力开发应用，而当前迫切需要创新具有新型配位单元和拓扑结构的MOF体系。其次，尽管多孔材料基吸附剂具有优异的吸附能力，但其对目标分析物的选择性仍有待提高。此外，通过对MOFs或COFs进行碳化处理得到的NPCs具备更优异的吸附能力，但其农药萃取方面的实际应用仍未得到充分探索。最后，我们对多孔材料基吸附剂应用潜力的调查表明，研究者更关注经典农药，而对当前环境中高频检出的农药以及新型农药的关注度不足。未来的研究方向应侧重于：（1）开发多样化的 NPCs，以充分发挥其结构优势；（2）探索生态友好型多孔材料，如氢键有机框架（HOF）和基于生物质/植物源单体的MOF/COF；（3）解决当前研究不平衡的问题，提高对新型和高频检出农药的关注度。
